# Supplementary material for: Global discovery of lupus genetic risk variant allelic enhancer activity
Source: Nat Commun. 2021 Mar 12;12:1611. doi: 10.1038/s41467-021-21854-5 (PMC7955039; doi:10.1038/s41467-021-21854-5)
Supplement: Supplementary file 1 — Supplementary Information [file 41467_2021_21854_MOESM1_ESM.pdf]

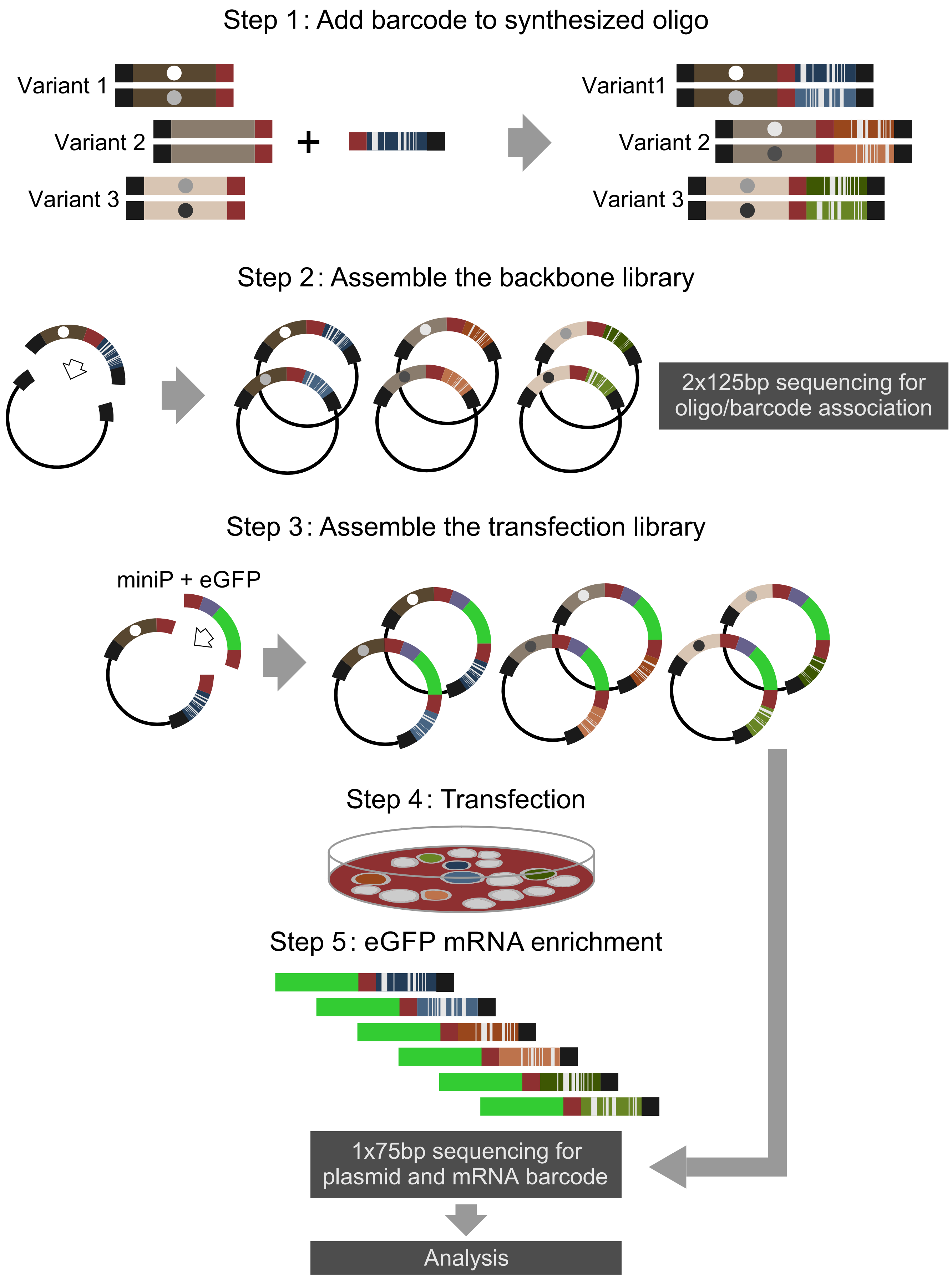

Supplementary Fig. 1. Massively Parallel Reporter Assay Workflow

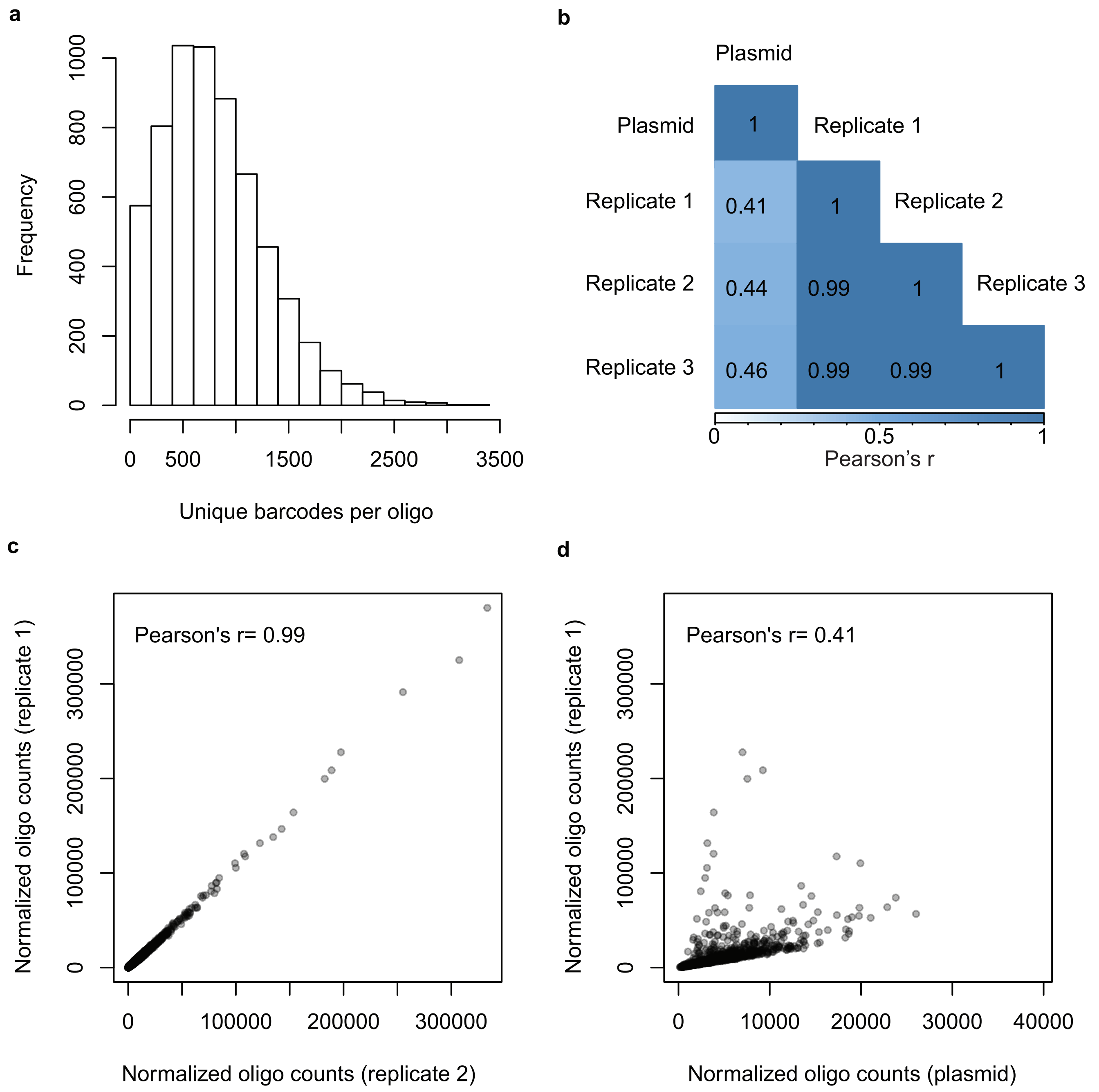

Supplementary Fig. 2. Quality assessment of the SLE MPRA library.
a. Histogram showing the distribution of unique barcodes associated with each oligo (allele of a SLE risk variant) in the plasmid library. b. The correlation between the normalized barcode count in the plasmid library and the RNA for three GM12878 MPRA experimental replicates. c. Scatter plot showing the correlation between the normalized barcode count in the RNA of GM12878 MPRA experimental replicates 1 and 2. d. Scatter plot showing the correlation between the normalized barcode count in the plasmid library and the RNA of GM12878 MPRA experimental replicate 1. Only data from enhancer alleles (enAlleles) are shown.

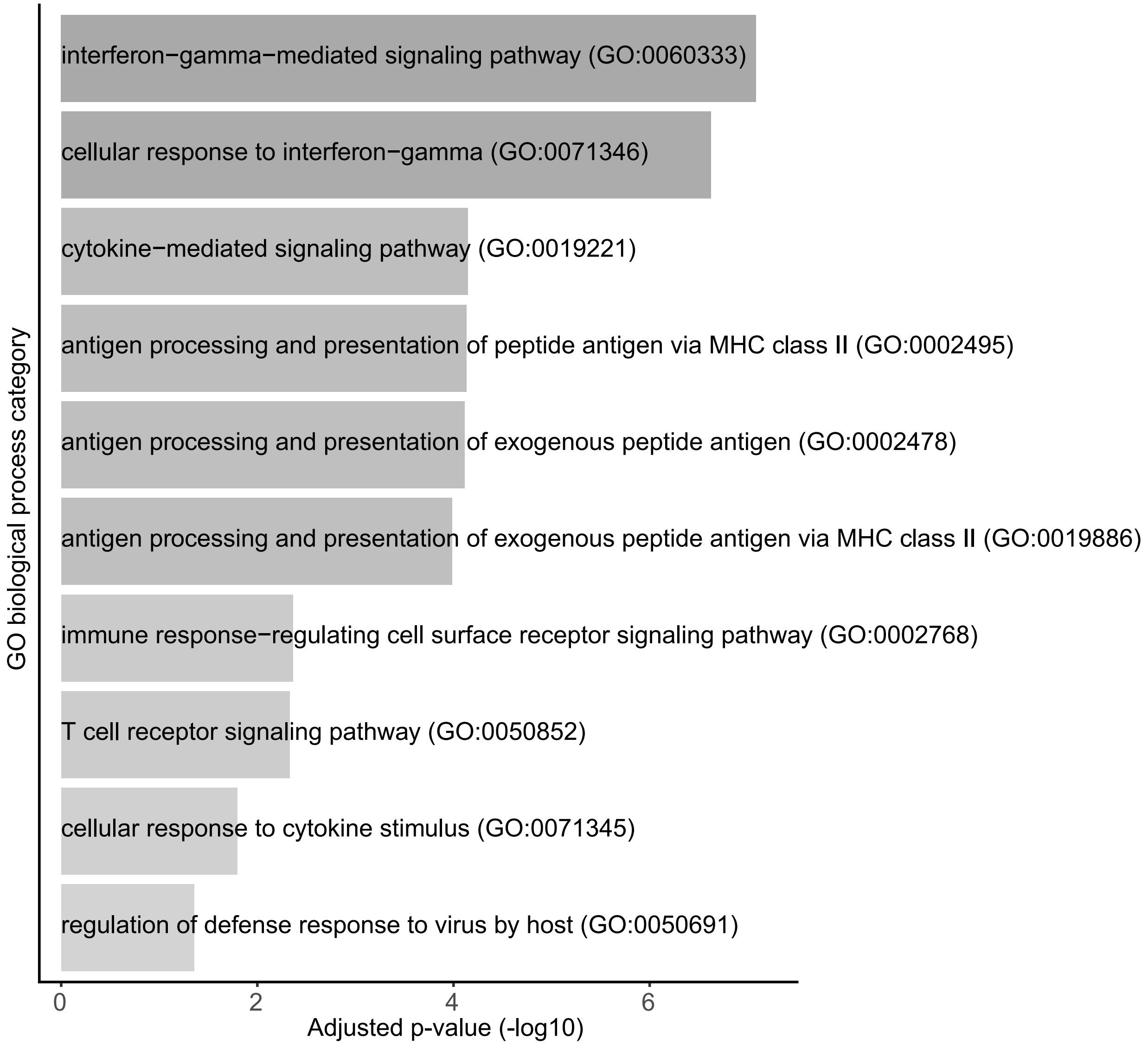

Supplementary Fig. 3. Pathway analysis of genes associated with enhancer variants (enVars).

Pathway enrichment analysis was performed with Enrichr using the predicted target genes of enVars in GM12878. The top 10 enriched pathways are shown. Dark grey bars are GO biological process enriched at a cut-off of p_{FDR} <0.05. The p-values were generated by two-sided Fisher's exact test with Benjamini-Hochberg multiple testing correction.

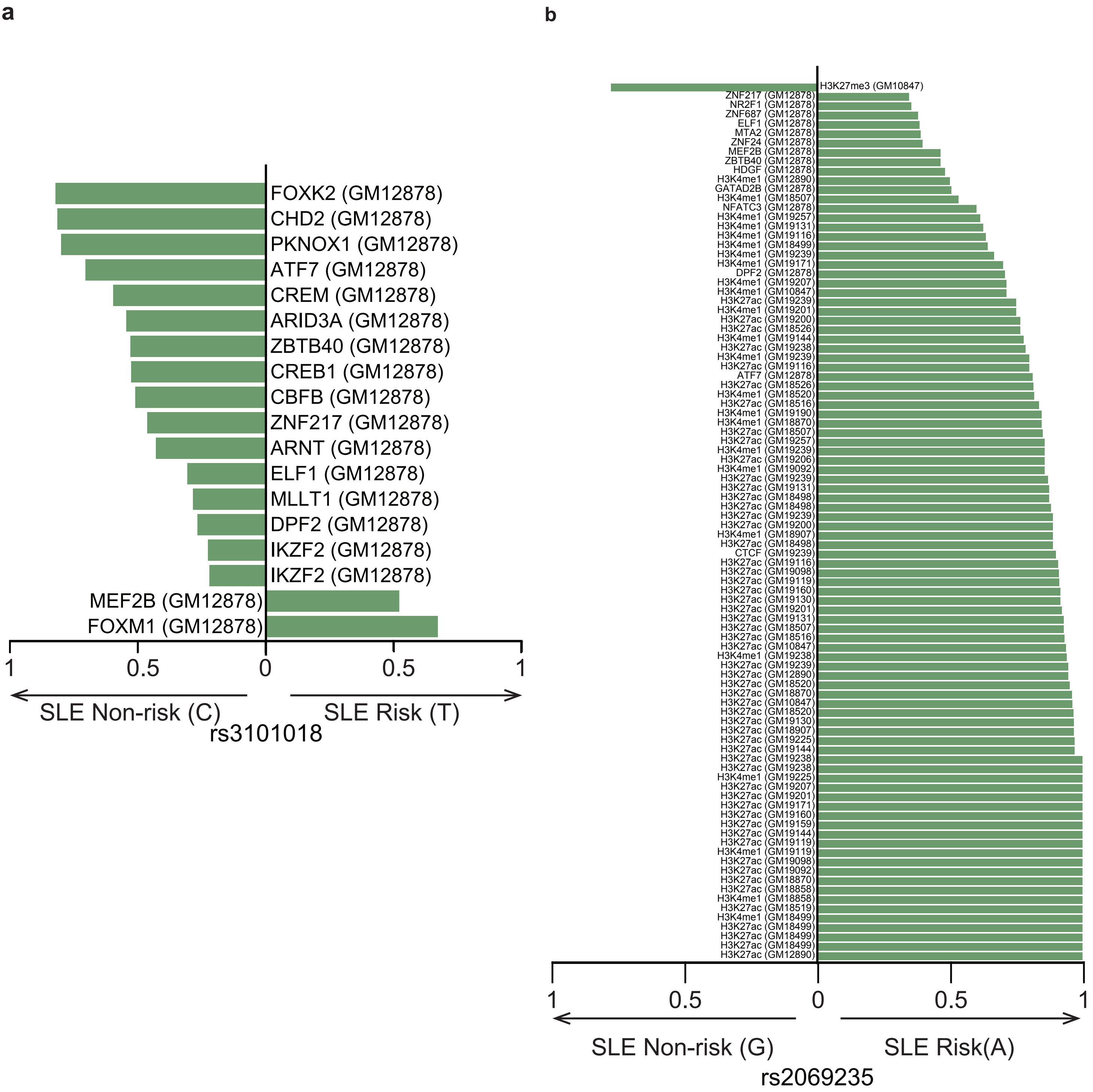

Supplementary Fig. 4. Genotype-dependent activity of transcription factors, transcriptional regulators, and histone marks in EBV-transformed B cell lines for rs3101018 and rs2069235.
a. and b. Results with MARIO ARS value >0.4 and consistent allelic imbalance across ChIP-seq datasets for rs3101018 and rs26069235 (see Methods). The X-axis indicates the preferred allele, along with a value indicating the strength of the allelic behavior, calculated as one minus the ratio of the weak to strong read counts (e.g., 0.5 indicates the strong allele has twice the reads of the weak allele).

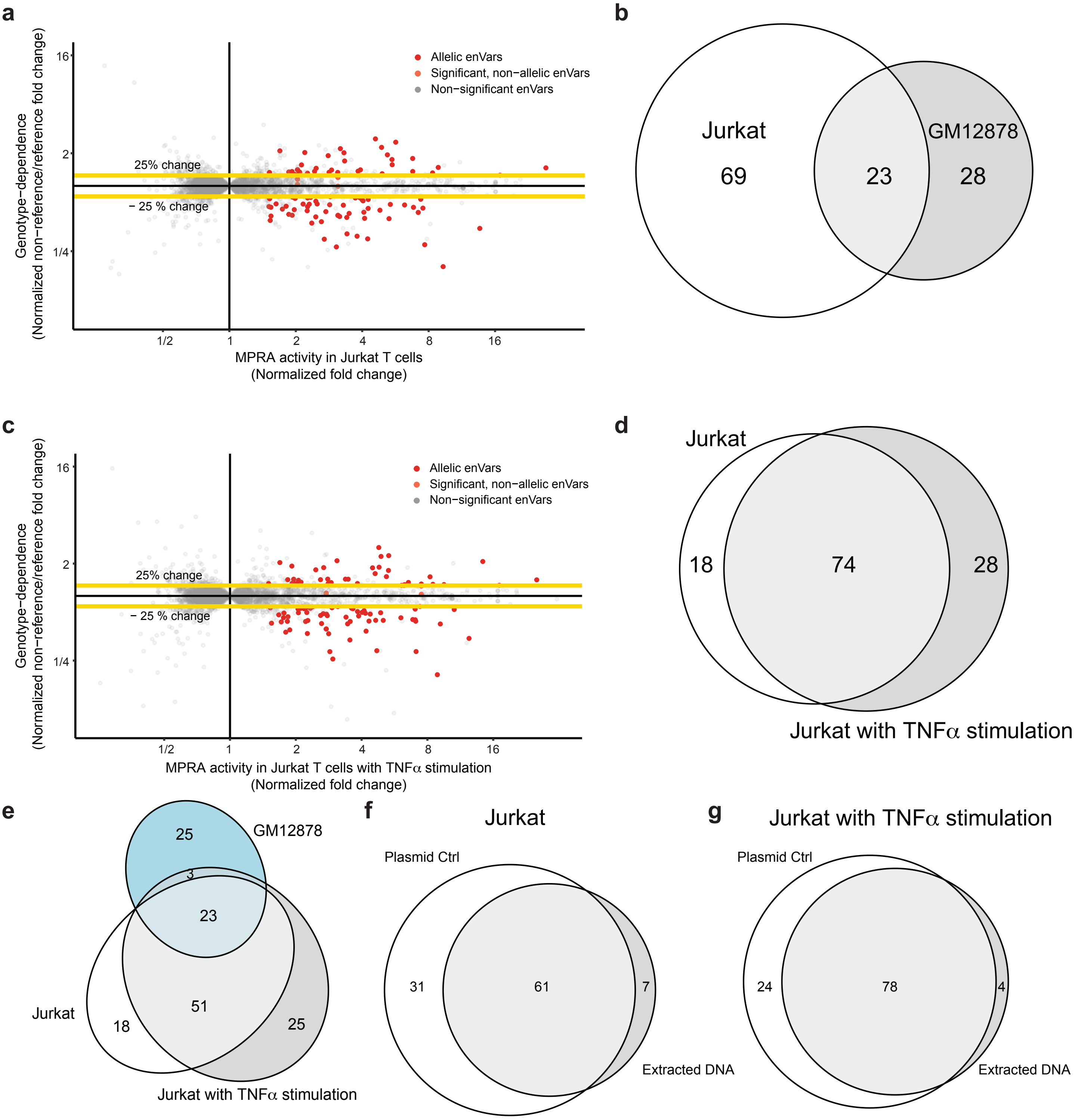

Supplementary Fig. 5. Regulatory activity of allelic enhancer variants in Jurkat T cells. a and c. Identification of allelic enhancer variants (enVars) in Jurkat cells and Jurkat cells with TNFα stimulation. Genotype dependence (Y-axis) is defined as the normalized fold change of MPRA activity between the non-reference and reference alleles (n=5 biologically replicates, see Methods). MPRA activity (X-axis) is presented as the maximum normalized fold-change of MPRA activity for any allele of the variant. Allelic enVars (red) were defined as variants with a significant difference in MPRA activity ($p_{adj} < 0.05$) between any pair of alleles and at least a 25% change in activity difference (see Methods). Plasmid control was used as control. The p-values were generated by two-sided Student's *t*-test with Benjamini-Hochberg multiple testing correction. b and d. Comparison of identified allelic enVars between Jurkat cells (white) and GM12878 cells (grey), and between Jurkat cells (white) and Jurkat cells with TNFα stimulation (grey). e. Comparison of identified allelic enVars among Jurkat cells (white), Jurkat cells with TNFα stimulation (grey), and GM12878 cells (blue). f and g. Comparison of identified allelic enVars in Jurkat cells and Jurkat cells with TNFα stimulation for different normalization approaches (see Supplementary Note 1). Normalization by plasmid control are shown in white. Normalization by extracted DNA are shown in grey.

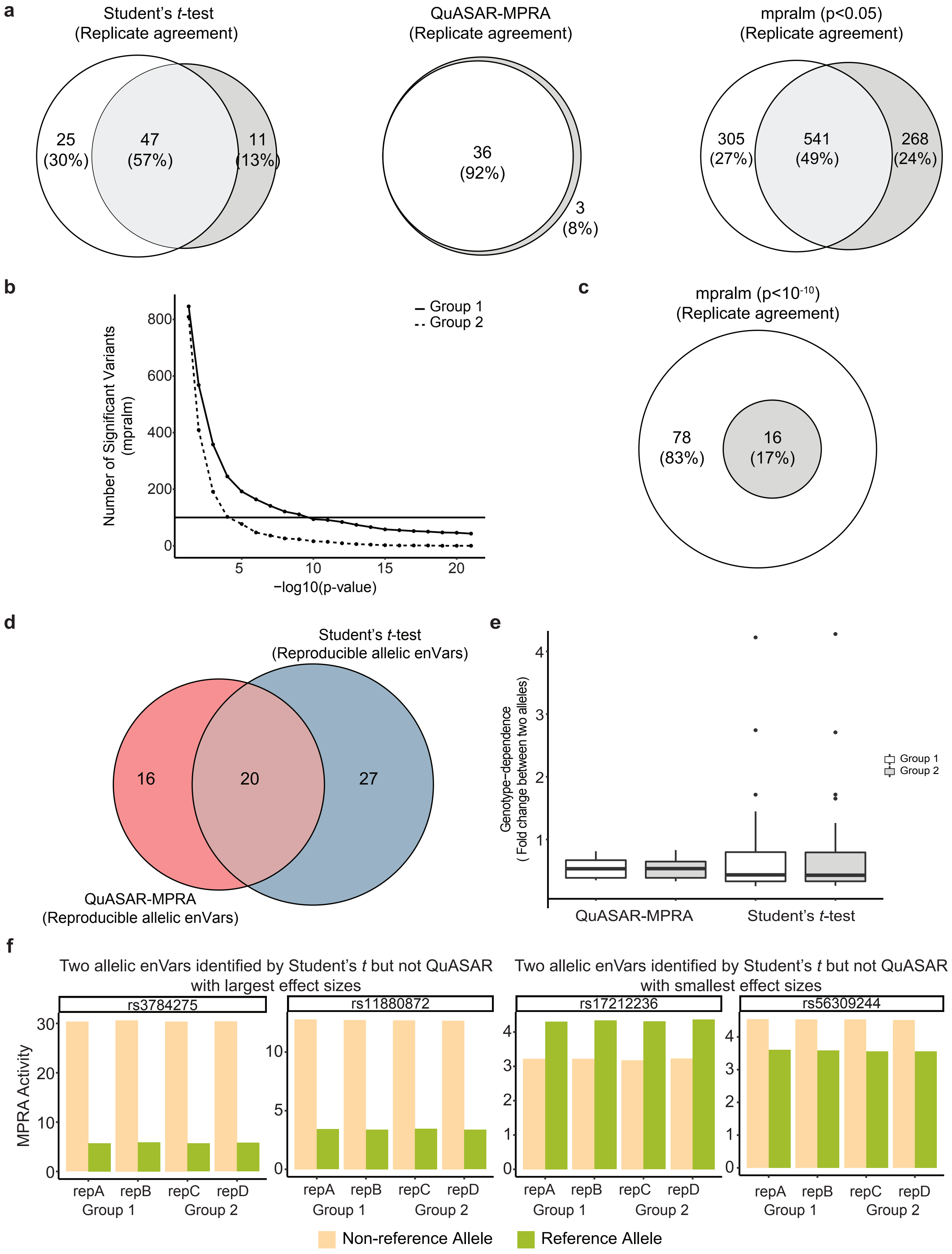
a
Student's t-test (Replicate agreement)
25 (30%)
47 (57%)
11 (13%)
QuASAR-MPRA (Replicate agreement)
36 (92%)
3 (8%)
mpralm (p<0.05) (Replicate agreement)
305 (27%)
541 (49%)
268 (24%)
b
Group 1
Group 2
Number of Significant Variants (mpralm)
−log10(p-value)
c
mpralm (p<10-10) (Replicate agreement)
78 (83%)
16 (17%)
d
Student's t-test (Reproducible allelic enVars)
16
20
27
QuASAR-MPRA (Reproducible allelic enVars)
e
Genotype−dependence (Fold change between two alleles)
Group 1
Group 2
QuASAR-MPRA
Student's t-test
f
Two allelic enVars identified by Student's t but not QuASAR with largest effect sizes
Two allelic enVars identified by Student's t but not QuASAR with smallest effect sizes
rs3784275
rs11880872
rs17212236
rs56309244
MPRA Activity
repA repB repC repD
Group 1 Group 2
Non-reference Allele
Reference Allele

Supplementary Fig. 6. Comparison of MPRA data analysis methods

a. Comparison of consistency across MPRA analysis methods. Venn diagrams indicate consistency of allelic enVars between Group 1 (white) and Group 2 (grey) replicate sets using three different MPRA data analysis methods (see Supplementary Note 2). b. Number of significant allelic enVars identified by mpralm in Group 1 (solid line) and Group 2 (dash line) at various mpralm p-value cut-offs. Horizontal line indicates 100 variants, which corresponds to a p-value cut-off of 10^{-4} and 10^{-10} in the two groups, respectively. c. Comparison of identified allelic enVars between Group 1 (white) and Group 2 (grey) at the $p<10^{-10}$ cut-off. d. Comparison of the allelic enVars identified in both Group 1 and Group 2 by the QuASAR-MPRA (red) and Student's *t*-test (blue) methods. e. Comparison of the strength of genotype dependence for allelic enVars uniquely identified by the QuASAR-MPRA (n=16 allelic enVars) and Student's *t*-test (n=27 allelic enVars) methods (see Supplementary Note 2). f. Normalized MPRA activity of replicates for rs3784275, rs11880872, rs17212236 and rs56309244. Group 1 is shown in yellow. Group 2 is shown in green. In e, data are represented as boxplots where the middle line is the median, the lower and upper hinges correspond to the first and third quartiles, the upper whisker extends from the hinge to the largest value no further than 1.5 × IQR from the hinge (where IQR is the inter-quartile range) and the lower whisker extends from the hinge to the smallest value at most 1.5 × IQR of the hinge, while data beyond the end of the whiskers are outlying points that are plotted individually. In a, d, e and f, p-value of Student's *t*-test was generated by two-sided Student's *t*-test with Benjamini-Hochberg multiple testing correction. p-value of QuASAR-MPRA was generated by beta-binomial model with Benjamini-Hochberg multiple testing correction. In a, b and c, p-value of mpralm was generated by linear model.

Supplementary Table 1 Primers used in this study

Vector Primer

Name	Sequence	Modification
Q5_deletion_rev	AGCTTGGCCGCCGAGGCCAG	None
Q5_deletion_fwd	AGTCGGGGCGGCCGGCCG	None
GFP_seq_MS2-P65-HSF1_GFP_FWD	CTGTTGGTAAAGCCACCATGGTGAGCAAGGGCGAGGAGCT	None
GFP_seq_MS2-P65-HSF1_GFP_REV	GCCGGCCGCCCCGACTCTAGAATTATTACTTGTACAGCTCGTCCATGCCG	None

Assemble Primer

Name	Sequence	Modification
200-MPRA_v3_GFP_Fusion_v2_F	CACTGCGGCTCCTGCGATCTAACTGGCCGGTACCTGAGCTCGCTA	None
201-MPRA_v3_GFP_Fusion_v2_R	TCTAGAGGTTCGTCGACGCGATTATTATCATTACTTGTACAGCTCGTCCATGC	None
MPRA_v3_F	GCCAGAACATTTCTCTGGCCTAACTGGCCGCTTGACG	None
MPRA_v3_201_R	CCGACTAGCTTGGCCGCCGACGCTCTTCCGATCT(N1:25252525)(N1)(N1)(N1)(N1)(N1)(N1)(N1)(N1)(N1)(N1)(N1)(N1)(N1)(N1)(N1)(N1)(N1)(N1)(N1)TCTAGAGGTTCGTCGACGCGATCGCAGGAGCCGCAGTG	None
MPRA_v3_Amp2Sc_R	CCGACTAGCTTGGCCGC	None
MPRA_V3_Illumina_GFP_F	GTGACTGGAGTTCAGACGTGTGCTCTTCCGATCTCGCCCTGAGCAAAGACC	None
MPRA_v3_TruSeq_Amp2Sa_F_P7	TGACTGGAGTTCAGACGTGTGCTCTTCCGATCTAACTGGCCGCTTGACG	None
TruSeq_Universal_Adapter_P5	AATGATACGGCGACCACCGAGATCTACACTCTTTCCCTACACGACGCTCTTCCGATCT	None

eGFP enrichment Primer

Name	Sequence	Modification
Biotin-labeled GFP probe One	CGCCGTAGGTCAGGGTGGTCACGAGGGTGGGCCAG/3BioTEG/	3' Biotin
Biotin-labeled GFP probe Two	CCAGGATGTTGCCGTCCTCCTTGAAGTCGATGCCC/3BioTEG/	3' Biotin
Biotin-labeled GFP probe Three	CCTCGATGTTGTGGCGGATCTTGAAGTTCACCTTG/3BioTEG/	3' Biotin

Library Index Primer

Name	Sequence	Modification
i701	CAAGCAGAAGACGGCATACGAGATCGAGTAATGTGACTGGAGTTCAGACGTGTGC	None
i702	CAAGCAGAAGACGGCATACGAGATTCTCCGGAGTGACTGGAGTTCAGACGTGTGC	None
i703	CAAGCAGAAGACGGCATACGAGATAATGAGCGGTGACTGGAGTTCAGACGTGTGC	None
i704	CAAGCAGAAGACGGCATACGAGATGGAATCTCGTGACTGGAGTTCAGACGTGTGC	None
i705	CAAGCAGAAGACGGCATACGAGATTTCTGAATGTGACTGGAGTTCAGACGTGTGC	None
i706	CAAGCAGAAGACGGCATACGAGATACGAATTCGTGACTGGAGTTCAGACGTGTGC	None
i707	CAAGCAGAAGACGGCATACGAGATAGCTTCAGGTGACTGGAGTTCAGACGTGTGC	None
i708	CAAGCAGAAGACGGCATACGAGATGCGCATTAGTGACTGGAGTTCAGACGTGTGC	None
i709	CAAGCAGAAGACGGCATACGAGATCATAGCCGGTGACTGGAGTTCAGACGTGTGC	None
i710	CAAGCAGAAGACGGCATACGAGATTTCGCGGAGTGACTGGAGTTCAGACGTGTGC	None
i711	CAAGCAGAAGACGGCATACGAGATGCGCGAGAGTGACTGGAGTTCAGACGTGTGC	None
i712	CAAGCAGAAGACGGCATACGAGATCTATCGCTGTGACTGGAGTTCAGACGTGTGC	None

Supplementary Note 1. Normalization control optimization

To our knowledge, two methods have been used to date as normalization controls for mRNA barcode counts: plasmids used for transfection[1] and DNA extracted from transfected cells[2]. We compared the allelic enVars that we identified through these two normalization methods using MPRA data from Jurkat cells with and without TNFα simulation. All MPRA data analysis steps were identical in this comparison, other than the use of plasmid or extracted DNA as the normalization control. As shown in Supplementary Fig. 5f, g**,** there is little difference in the results produced by the two different normalization strategies. Moreover, the plasmid control strategy identified slightly more allelic enVars in both experiments. We conclude from these comparisons that the plasmid control is an effective, and slightly more sensitive normalization approach.

Supplementary Note 2. Comparison of MPRA data analysis methods

To date, four methods have been used to our knowledge for enAllele/enVar and allelic enVar identification: DESeq2 combined with Student's *t*-test[1, 3], QuASAR-MPRA[4], mpralm[5] and MPRAnalyze[6]. For the DESeq2 combined with Student's *t*-test method, we followed the analysis procedure in Tewhey *et al.*[1], with the application of additional more stringent criteria, as described in the "Enhancer variant (EnVar) identification" and "Allelic enVar identification" sections of the Methods. For the other three methods, we followed the analysis procedures described in the corresponding publications.

To compare the performance of these four methods, we developed approaches to compare their (1) specificity, (2) consistency, and (3) sensitivity. We note that this analysis was hindered somewhat due to the lack of a reliable gold standard. First, we combined all of our GM12878 experimental replicate data and plasmid control data and re-sampled them into four replicate sets (repA, repB, repC, and repD), respectively. We then grouped repA and repB into Group 1 and repC and repD into Group 2. Next, we ran each of the four analysis methods to identify enAlleles and allelic enVars in Group 1 and Group 2. We expected to find a small number of differences between the results of Group 1 and Group 2, since they are sampled from the same set of reads.

Of the four analysis methods, DESeq2 combined with Student's *t*-test, QuASAR-MPRA and mpralm methods ran successfully in our hands. The MPRAnalyze pipeline failed to run and had extensive computational resource requirements - the program ran with 200GB of RAM for >17hrs, with no successful completion in three attempts.

To assess specificity, we compared the Group 1 and Group 2 enAllele predictions. Both the DESeq2 combined with Student's *t*-test and mpralm methods found no significant differences between the subsampled groups, indicating strong specificity (and also further highlighting the

reproducibility of our data). QuASAR-MPRA does not have a function to perform enhancer identification, so we could not gauge its specificity using this procedure.

To assess consistency, we identified allelic enVars in Group 1 and Group 2, respectively and used a $p<0.05$ allelic cut-off. From a total of 3,093 variants, the DESeq2 combined with Student's *t*-test method identified a total of 83 allelic enVars in the two groups, with 57% in common between Group 1 and Group 2. QuASAR-MPRA identified 39 allelic enVars, with 92% in common between Group 1 and Group 2. mpralm identified 1,114 allelic enVars, with 49% in common between Group 1 and Group 2 (Supplementary Fig. 6a). 1,114 allelic enVars is 36% of all tested variants, which indicated to us that a more stringent p-value cut-off was needed for mpralm. The number of allelic enVars dropped exponentially at more stringent cut-offs (Supplementary Fig. 6b). Based on this analysis, we chose a cut-off $p<10^{-10}$, which results in 94 allelic enVars in the two groups (a comparable number to that identified by the other methods). At this threshold, only 17% of the mpralm-identified allelic enVars were shared in both groups (Supplementary Fig. 6c). Additionally, because Group 1 and Group 2 were sampled from the same pool, it was concerning that mpralm consistently identified more significant allelic enVars from Group 1 compared to Group 2 despite re-running of the code numerous times by two different people. This inconsistency combined with the inflated number of identified allelic enVars at reasonable p-value cutoffs greatly reduced our enthusiasm for mpralm.

Considering the poor performance of mpralm, we subsequently focused on the DESeq2 combined with Student's *t*-test and QuASAR-MPRA methods. We first compared the final allelic enVar predictions of these two methods (i.e. those predicted by the given method to be allelic in both Group 1 and Group 2). As shown in Supplementary Fig. 6d, DESeq2 combined with Student's *t*-test identified 27 unique variants, while QuASAR-MPRA identified 16 unique variants. The unique QuASAR-MPRA variants tended to have a larger variance between replicates, diminishing our confidence in these results. Variants unique to the DESeq2 combined with Student's *t*-test method

tend to have a much wider range of genotype-dependence values (Supplementary Fig. 6e), indicating that this method is likely more sensitive. To further explore sensitivity, we manually examined raw MPRA data for four unique Student's *t*-test variants (the two variants with the largest amount of genotype-dependence and the two with the smallest). As shown in Supplementary Fig. 6f, all four variants show consistent allelic difference among the replicates in both groups, raising questions about why QuASAR-MPRA did not detect them. Taken together, these results suggest to us that the DESeq2 combined with Student's *t*-test method currently represents the analytical strategy with the best balance between specificity, consistency, and sensitivity.

Supplementary References

1. Tewhey R*, et al.* Direct Identification of Hundreds of Expression-Modulating Variants using a Multiplexed Reporter Assay. *Cell* **165**, 1519-1529 (2016).

2. Klein JC*, et al.* Functional testing of thousands of osteoarthritis-associated variants for regulatory activity. *Nat Commun* **10**, 2434 (2019).

3. Ray JP*, et al.* Prioritizing disease and trait causal variants at the TNFAIP3 locus using functional and genomic features. *Nat Commun* **11**, 1237 (2020).

4. Kalita CA, Moyerbrailean GA, Brown C, Wen X, Luca F, Pique-Regi R. QuASAR-MPRA: accurate allele-specific analysis for massively parallel reporter assays. *Bioinformatics* **34**, 787-794 (2018).

5. Myint L, Avramopoulos DG, Goff LA, Hansen KD. Linear models enable powerful differential activity analysis in massively parallel reporter assays. *BMC Genomics* **20**, 209 (2019).

6. Ashuach T, Fischer DS, Kreimer A, Ahituv N, Theis FJ, Yosef N. MPRAnalyze: statistical framework for massively parallel reporter assays. *Genome Biol* **20**, 183 (2019).
