# Supplementary material for: Global discovery of lupus genetic risk variant allelic enhancer activity
Source: Nat Commun. 2021 Mar 12;12:1611. doi: 10.1038/s41467-021-21854-5 (PMC7955039; doi:10.1038/s41467-021-21854-5)
Supplement: Supplementary file 2 — Description of Additional Supplementary Files [file 41467_2021_21854_MOESM2_ESM.pdf]

Description of Additional Supplementary Files

File Name: Supplementary Data 1
Description: GWAS Loci List. List of all the SLE associated tag genetic variants reaching genome-wide significance published through March 2018. This list was used for linkage disequilibrium expansion.

File Name: Supplementary Data 2
Description: Summarized Information for all the variants. Information about the genetic variants examined in this study.

File Name: Supplementary Data 3
Description: DESeq2 results for calibration variants. This table summarizes the DESeq2 results for the calibration variants used in this study in GM12878. It contains both results from current study and previous study (PMID: 27259153). These variants are only used for calibration purpose. The raw result and confusion matrix are included on two separate sheets.

File Name: Supplementary Data 4
Description: Sequence information for all the variants. The sequence of each oligo used in this study.

File Name: Supplementary Data 5
Description: Raw MPRA count data at the allele/oligo level. The summarized count of unique barcodes for each oligo in this study.

File Name: Supplementary Data 6
Description: DESeq2 results for all variants. The raw output from DESeq2 at the allele/oligo level. DESeq2 is used to compare MPRA experimental replicates vs plasmid control to identify alleles/oligos with enhancer activity. Only alleles/oligos with more than 30 unique barcodes are included for analysis.

File Name: Supplementary Data 7
Description: Enhancer variant (enVar) promoter looping genes and eQTL gene annotation. The looping data contains promoter looping genes related to the variant in the GM12878 cell. The eQTL data contains the eQTL genes for the variant in available cell sources, including GM12878.

File Name: Supplementary Data 8
Description: GO biological process enrichment results. GO biological process enrichment results for the target genes of enhancer variants (enVars) in GM12878. The analysis was performed with Enrichr (see Methods). The target genes of enVars are the combined genes for enVars in the All_Target_Gene column of Supplementary Data 2.

File Name: Supplementary Data 9
Description: RELI results for enhancer variants (enVars) in GM12878. Raw output for RELI analysis of GM12878 enVars using GM12878 ChIP-seq datasets. The result is separated into transcription factor results and histone modification result.

File Name: Supplementary Data 10
Description: HOMER TF motif enrichment results for enhancer alleles (enAlleles) in GM12878. Raw output for HOMER analysis of GM12878 enhancer alleles (enAlleles).

File Name: Supplementary Data 11
Description: Student's t-test results for enhancer variants (enVars). Raw output for Student's t-test results for enhancer variants (enVars).

File Name: Supplementary Data 12
Description: Annotation of allelic enVars in GM12878.

File Name: Supplementary Data 13
Description: RELI results of allelic enhancer variants (allelic enVars) in GM12878. Raw output for RELI analysis of allelic enVars in GM12878 ChIP-seq data.

File Name: Supplementary Data 14
Description: Allele-dependent analysis using the MARIO pipeline. This table summarizes the results of MARIO allelic analyses for GM12878 allelic enVars. We applied our previously developed and published MARIO method for detecting allele-dependent sequencing reads in ChIP-seq (and related) datasets. We focused on the group of allelic enVars we identified through MPRA that are heterozygous in the GM12878 cell line or other available lymphoblastoid cell lines (LCLs) (this is a requirement, because both alleles need to be present). Allele-dependent reads in all publicly available LCL data were then calculated for this variant group. Results were combined across replicates, and the final MARIO Allelic Reproducibility Score (ARS) was calculated for each variant and ChIP-seq dataset pair.

File Name: Supplementary Data 15
Description: Full results of variant overlapping and variant adjacent TFs analyses. Results of GM12878 allelic enVar TF analysis. The "variant overlapping" worksheet includes the motif disruption analysis proportion test results. The "variant adjacent" worksheet includes the motif enrichment analysis proportion test results.
